# Scanning electron microscopy of ibrutinib-induced hair shaft changes^☆^

**DOI:** 10.1016/j.abd.2022.10.005

**Published:** 2023-03-02

**Authors:** Hiram Larangeira de Almeida Jr., Debora Sarzi Sartori, Douglas Malkoun, Carlos Eduardo Pouey Cunha

**Affiliations:** a Department of Dermatology, Universidade Federal de Pelotas, Pelotas, RS, Brazil; b Graduate Program in Health and Behavior, Universidade Católica de Pelotas, Pelotas, RS, Brazil

Dear Editor,

Bruton's tyrosine kinase (BTK) is essential for the development and maturation of B-lymphocytes. These cells do not mature in individuals with mutations in this enzyme, and they have X-linked agammaglobulinemia, the most common type of congenital agammaglobulinemia.^1^

BTK is also expressed in tumor cells, and its inhibition is gaining increasing importance in the treatment of B-lineage neoplasms.^2^ BTK participates in the activation of these cells, being important for the survival of malignant B cells and, therefore, its inhibition decreases their proliferation and survival.

Ibrutinib is a potent irreversible BTK inhibitor and was the first inhibitor of this enzyme approved by the FDA for the treatment of the following diseases in adults: 1) Chronic graft-versus-host disease, after the failure of one or more systemic therapy lines; 2) Chronic lymphocytic leukemia/small cell lymphocytic lymphoma (CLL/SCLL); 3) CLL/SCLL in adults with 17p deletion; 4) Mantle-cell lymphoma in adults who have received at least one prior therapy; 5) Relapsed/refractory marginal zone lymphoma, in adults who require systemic therapy and have received at least one prior anti-CD20-based therapy; and 6) In Waldenström's macroglobulinemia.^3^

The described side effects are fatigue, diarrhea, peripheral edema, cardiac arrhythmia (atrial fibrillation), bleeding, and infections (respiratory tract).^4^, ^5^, ^6^

Ibrutinib can have cutaneous adverse effects, with a peak incidence in the first year of treatment. The most common manifestations are skin rash, petechiae and ecchymosis. Also, urticaria, herpes simplex and herpes zoster reactivation, panniculitis, and Stevens-Johnson syndrome may occur.^6^, ^7^

Despite the specificity for BTK, some skin effects are similar to those produced by EGF (epidermal growth factor) inhibitors,^8^ such as the described acneiform rash, changes in hair/eyelashes,^9^ and longitudinal grooves on nails.

A 72-year-old patient who had received ibrutinib for six months to treat mantle lymphoma unresponsive to conventional therapy was assessed. The patient reported a slight alteration in his hair, with a change in its curling (Fig. 1). Some hair shafts were cut and examined *in natura* with scanning electron microscopy. At medium magnification, discreet longitudinal channels were observed in the hair shafts (Fig. 2A and B), which are not seen in normal hair (Fig. 2C). At high magnification, these channels were very evident (Figure 3, Figure 4).

**Figure 1:**
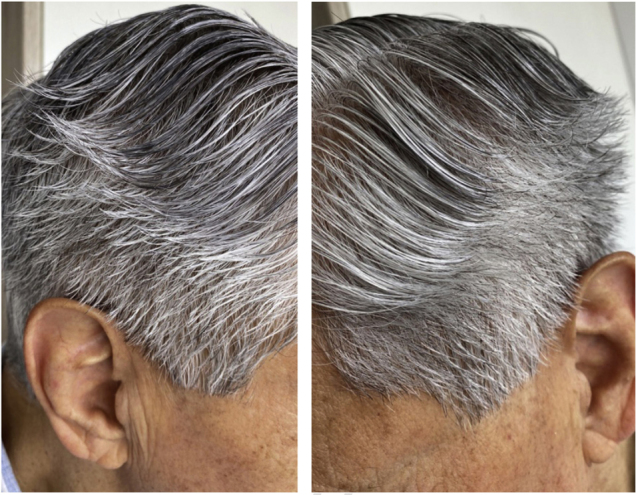
Modification in hair curl pattern.

**Figure 2:**
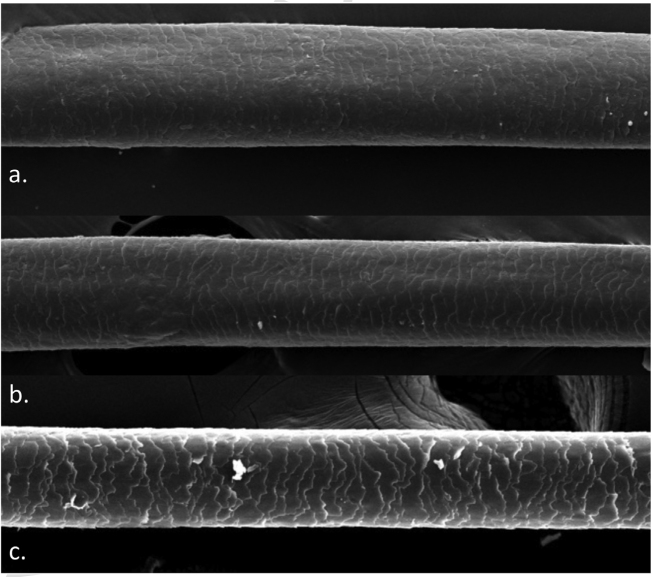
Scanning electron microscopy. (A. and B) Medium magnification showing discreet hair shaft channels (×300 and 270). (C) normal control (×300).

**Figure 3:**
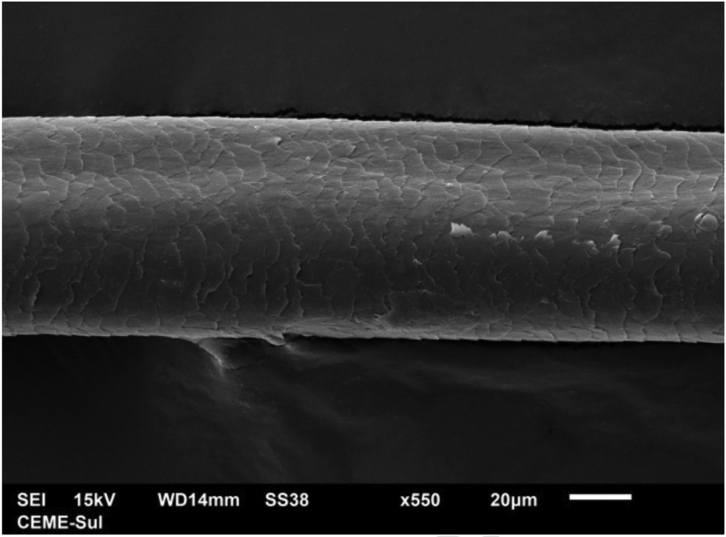
Scanning electron microscopy. High magnification revealing a channel in the hair shaft (×550).

**Figure 4:**
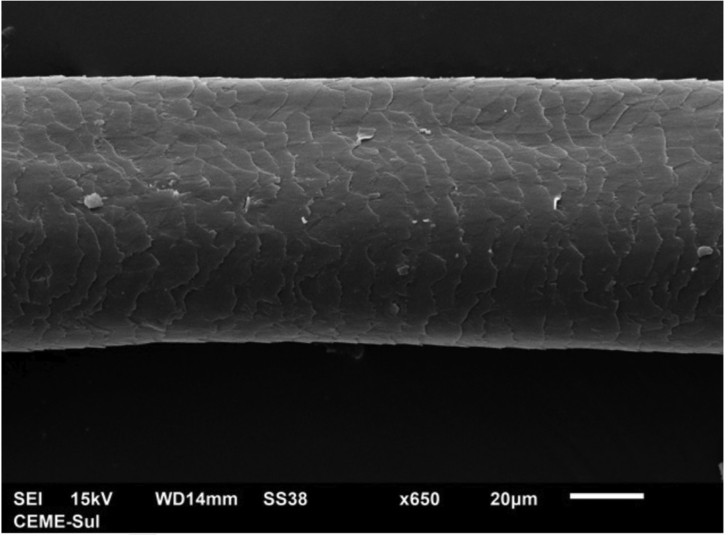
Scanning electron microscopy. High magnification showing a channel in the hair shaft (×650).

The authors did not find any reports in the literature of an examination of hairs shafts with scanning electron microscopy showing alterations caused by ibrutinib. There are some reports with EGF inhibitors, in which channels were also described in the hair shafts,^9^ in this case with greater clinical consequences, making the hair frizzy and the eyelashes elongated (trichomegaly) and without curvature. The channels seen in these drug-induced cases are similar to those seen in families with uncombable hair,^10^ and in a syndromic type of pili canaliculi associated with a central nervous system degeneration called giant axonal neuropathy.^11^

The findings of this patient demonstrate, on ultrastructural examination, a similarity between the alterations of EGF and BTK inhibitors.

BTK inhibition is an expanding concept in the treatment of hematologic malignancies, with 22 drugs under development, and the emergence of drugs with less systemic and cutaneous toxicity is possible in the near future.^10^, ^12^

## Financial support

None declared.

## Authors' contributions

Hiram Larangeira de Almeida Jr: Approval of the final version of the manuscript; design and planning of the study; drafting and editing of the manuscript; collection, analysis, and interpretation of data; effective participation in research orientation; intellectual participation in the propaedeutic and/or therapeutic conduct of the studied cases; critical review of the literature; critical review of the manuscript.

Debora Sarzi Sartori: Approval of the final version of the manuscript; design and planning of the study; drafting and editing of the manuscript; collection, analysis, and interpretation of data; intellectual participation in the propaedeutic and/or therapeutic conduct of the studied cases; critical review of the literature; critical review of the manuscript.

Douglas Malkoun: Approval of the final version of the manuscript; design and planning of the study; drafting and editing of the manuscript; collection, analysis, and interpretation of data; intellectual participation in the propaedeutic and/or therapeutic conduct of the studied cases; critical review of the literature; critical review of the manuscript.

Carlos Eduardo Pouey Cunha: Approval of the final version of the manuscript; design and planning of the study; drafting and editing of the manuscript; collection, analysis, and interpretation of data; intellectual participation in the propaedeutic and/or therapeutic conduct of the studied cases; critical review of the literature; critical review of the manuscript.

## Conflicts of interest

None declared.
